# Supplementary material for: Puzzles in modern biology. II. Language, cancer and the recursive processes of evolutionary innovation
Source: F1000Res. 2016 Sep 9;5:2289. [Version 1] doi: 10.12688/f1000research.9568.1 (PMC5288677; doi:10.12688/f1000research.9568.1)
Supplement: Peer review history

# Peer review: Referee response for version 1

**DOI:** 10.5256/f1000research.10305.r17054

**Published:** 2016-12-06

**Authors:** Jon Wilkins

**Affiliations:** 1 Ronin Institute, Montclair, NJ, USA

The most easily understood mode of evolution is the one where small, incremental changes accumulate over very long timescales, leading to gradual changes in form and function that can be observed in the continuity of forms in the fossil record. However, it is also apparent that, on occasion, there are rapid bouts of change and diversification. On the timescale that we normally think about for evolutionary processes, these changes can seem virtually instantaneous.

 Steven Frank illustrates this sort of abrupt evolutionary change with two examples: the diversification of flowering plants and the development of human language. In both cases, the “why” and “how” of the sudden transformations remain poorly understood. 

 The key insight of this paper is that cancer evolution — involving natural selection acting on clones within a developing tumor — is another example of rapid evolutionary innovation. But unlike the other examples, which represent ancient, and perhaps unique, events, tumor development is a process that is happening again and again today, on a timescale that is amenable to study using a variety of tools.

 Of course, understanding cancer progression is important in its own right. But this piece point out that the insights coming from the study of cancer may help us to understand other, less accessible, evolutionary transformations. 

 Frank suggests that the key feature that permits this rapid change is “recursion”, which is used in a strong and weak sense — both of which are somewhat different from the way the term is typically employed in linguistics. In the weak sense, any phenomenon of exponential growth is deemed recursive in the sense that the outputs of this generation’s reproduction serve as the inputs for the next generation. He makes the point that exponential growth can create the illusion of suddenness when our observations are at a fixed scale.

 However, all biological reproduction is inherently exponential, and this argument should apply equally to the sudden evolutionary innovations and the more gradual transformations. So it is not clear why this would be the critical attribute for bursts of innovation. 

 Somewhat more compelling is the stronger version of recursion used here, where one innovation actually changes the landscape for future innovations. This type of innovative potentiation is more clearly a hallmark of abrupt evolutionary transformation, and there is potentially great insight to be had from recognizing the commonalities among seemingly diverse types of rapid innovation.

I have read this submission. I believe that I have an appropriate level of expertise to confirm that it is of an acceptable scientific standard.

# Peer review: Referee response for version 1

**DOI:** 10.5256/f1000research.10305.r16214

**Published:** 2016-09-13

**Authors:** Andy Gardner

**Affiliations:** 1 School of Biology, University of St Andrews, St Andrews, UK

The puzzle considered here is rapid evolutionary innovation, and Steven Frank draws parallels between the evolution of cancers and the evolution of language. Cancerous cell lineages must rapidly innovate in highly sophisticated ways to avoid being killed off by the immune system, and to achieve continued growth in an environment that is rapidly changing – including as a consequence of their own behaviour. Human language and associated cultural innovations appear to have happened in an explosive way that some have suggested is inconsistent with gradual evolutionary change.

 Frank suggests possible commonalities between these two phenomena, including how recursive growth may involve a ‘long fuse’ phase at which key innovation is occurring – perhaps invisibly – well before the explosion takes place, and how recursion itself may facilitate innovation owing to feedback of input and output.

 This is a compelling synthesis that immediately suggests many more questions, and I’m sure will stimulate further investigation and new insights.

 This article might benefit from the addition of a figure: an illustration or schematic showing how recursion facilitates explosive, innovative evolution. Also, for comparative purposes, might certain instances of stagnation and stasis be conceptualised and explained in terms of relative lack of recursion?

 As is my policy, I waive anonymity.

 Andy Gardner

I have read this submission. I believe that I have an appropriate level of expertise to confirm that it is of an acceptable scientific standard.
